# Clinical Genetic Aspects of ASD Spectrum Disorders

**DOI:** 10.3390/ijms17020180

**Published:** 2016-01-29

**Authors:** G. Bradley Schaefer

**Affiliations:** University of Arkansas for Medical Sciences, Arkansas Children’s Hospital, 1 Children’s Way, Slot 512-22, Little Rock, AR 72202, USA; schaefergb@uams.edu; Tel.: +1-501-364-2971; Fax: +1-501-364-1564

**Keywords:** multifactorial inheritance, genetic testing, diagnostic yield, copy number variants, gene sequencing, genomics

## Abstract

Early presumptions opined that autism spectrum disorder (ASD) was related to the rearing of these children by emotionally-distant mothers. Advances in the 1960s and 1970s clearly demonstrated the biologic basis of autism with a high heritability. Recent advances have demonstrated that specific etiologic factors in autism spectrum disorders can be identified in 30%–40% of cases. Based on early reports newer, emerging genomic technologies are likely to increase this diagnostic yield to over 50%. To date these investigations have focused on etiologic factors that are largely mono-factorial. The currently undiagnosed causes of ASDs will likely be found to have causes that are more complex. Epigenetic, multiple interacting loci, and four dimensional causes (with timing as a variable) are likely to be associated with the currently unidentifiable cases. Today, the “Why” is more important than ever. Understanding the causes of ASDs help inform families of important issues such as recurrence risk, prognosis, natural history, and predicting associated co-morbid medical conditions. In the current era of emerging efforts in “personalized medicine”, identifying an etiology will be critical in identifying endo-phenotypic groups and individual variations that will allow for tailored treatment for persons with ASD.

## 1. Introduction

### 1.1. Autism as a Neuro-Genetic Disorder

Not long after autism was identified in the 1940s as a distinct developmental disorder, the question of causation was discussed. Most of the early theories as to the etiology of autism were framed in the context of the prevailing psychiatric models of the time. Thus many theorized that autism was an acquired condition associated with children raised by cold, emotionally-distant mothers. Given our current understanding of the etiology of autism spectrum disorders (which will be a large focus of this review) it is hard to imagine how these theories gained popular acceptance. One has to also appreciate the extreme guilt that this must have evoked on the parents, and this compounded by the stress of having a special needs child.

From the 1940s to the present, the understanding of the etiology of autism has mirrored the eras of scientific advancement in the field of developmental biology and genetics. Population studies in the 1960s and 1970s provided clear proof of the biologic and genetic basis of autism. Progress in genetic testing in the 1980s and 1990s began to identify specific genetic markers in some patients with autism. The progression from linkage studies to cytogenetic, molecular cytogenetic, molecular and now genomic testing have all been very congruent in identifying genomic “hot spots” associated with autism.

Current understanding recognizes autism as having a strong genetic basis with a complex inheritance pattern. Strong genetic factors are involved. As with all human medical conditions, there is environmental modulation. There is clear etiologic and genetic heterogeneity. Literally hundreds of “autism genes” have been identified. Thus, from an etiologic standpoint, it would be better stated “the autisms” rather than “autism”. This understanding will be critical as the science of autism therapies moves forward. Using targeted therapies for specific identified causes of autism holds the promise of improved outcomes and reduced adverse events. For the purposes of this review I will discuss autism spectrum disorders (ASDs) unless otherwise stated. In general, from a clinical genetic standpoint, the evaluation is not different for persons on different parts of the spectrum. The focus of this review is to highlight the genetic factors in autism especially in the light of clinical applications.

#### 1.1.1. Indisputable Evidence of the Genetic Basis of Autism Spectrum Disorders (ASDs)

As noted above, population studies have provided strong evidence of the genetic basis of ASDs [1]. In fact, when compared to other neurodevelopmental disabilities (NDD), autism is one of most—if not the most—heritable NDDs known.

From 2006 to 2008 we reviewed a large portion of the existing published information on the population genetic characteristics of autism spectrum disorders [2,3]. This information provided a very strong indication of the genetic basis of ASDs. Classic parameters of population genetics that supported this included data on twin concordance, heritability, relative risk ratio and sibling risk ratio.

In this review, I will provide a brief summary of this collected body of information. Twin concordance studies showed an estimated 70% concordance among monozygotic twins (reported ranges 36%–95%). In contrast, concordance among dizygotic twins was around 3% (range 0%–31%) or 30% if a broader phenotypic definition was used. Heritability estimates were noted to be 0.8 to 0.9. The estimated relative (sibling) risk ratio is reported to be about 150 for monozygotic twins and 8–10 for dizygotic twins and full siblings [4]. In the realm of the discipline of population genetics this is overwhelming support of a genetic basis to ASDs. These data have been replicated and validated in more recent and larger studies [4,5,6]. With a broader design and larger sample size, these more recent studies have also added another population parameter not previously noted. A 2–3-fold increased recurrence risk of ASD has been documented in half-siblings (both maternal and paternal) of probands with an ASD [4,5,6,7]. The increased occurrence in even less closely related individuals lends very strong support for the genetic basis of autism.

#### 1.1.2. Why Then Is the Genetic Basis of ASDs Still Debated?

Even with such a strong body of evidence as to the genetic basis of ASDs, not everyone is convinced. Significant opposition to this notion exists. There exist multiple factions of people who do not recognize the validity of the commonly accepted medical literature. Common objections voiced include the concern over some link between ASDs and childhood immunizations. Some question how could a “genetic disorder” be increasing in frequency? This is cited in light of the continued increase in the reported incidence of ASD. Over the past 20 years the reported occurrence of ASD has almost quadrupled [8]. Current estimates put the prevalence of ASD at about 1/68 children [9]. The logical question is “How could a condition could be increasing at such a rapid rate and yet have a primary genetic origin?” As one of my partners expressed to me “It is interesting that the incidence of conditions like diabetes and asthma are similarly increasing and yet no one is questioning the genetic basis of these conditions [10].

### 1.2. Multifactorial Inheritance

Strictly speaking, all human medical conditions could be classified as having a “multi-factorial etiology”. This implies that these conditions have both genetic and environmental factors which contribute to the overall phenotype. All conditions have genetic and environmental contributions—depending on the condition, the relative proportion of each varies considerably. *Mendelian inheritance* is the term applied to those conditions in which a single gene mutation has a major phenotypic effect with significantly less environmental influences on the phenotype and in which the inheritance follows a monogenic pattern. For more complex traits, the relative contribution of environmental and genetic factors is more balanced. Conditions are designated as having *multifactorial inheritance* if several characteristics are noted:

1. Clear genetic variability exists yet no uni-factorial mode of inheritance can be identified
2. Family studies indicate an increased risk for near relatives to be affected
3. Complicated pathophysiology or morphogenetic processes are involved
4. Biologic influences of environmental factors

Genetic susceptibility refers to a condition that is predominantly determined by environmental factors with expression and phenotypic variability affected by genetic changes that alter the susceptibility to the environmental factor [11].

Considering the above definitions, ASDs clearly fall into the category of multi-factorial inheritance. The recurrence risk pattern of multi-factorial traits typically demonstrates:

1. An increased recurrence risk in close relatives as compared to the general population frequency
2. A non-linear decrease in frequency with increasing distance of relationship—typically no increased recurrence rates are seen beyond 3rd degree relatives
3. The recurrence risk increases with the number of affected individuals
4. There is an increased risk with increased severity of the condition
5. There is an increased risk if person(s) affected are of the “rarer” gender. (In ASD a distinct gender bias of a 3- to 4-fold rate of affected males has been noted)

Recurrence risk data that had accumulated up until about 2008 matched well with this model. The overall recurrence risk for a sibling of a single proband with ASD was reported as 3%–10%. Further refinement of these risks noted that the estimated recurrence risk for a sibling of an affected person with an ASD was established as 4% if the affected individual was male and 7% if female. If there were more than one affected sibling with an ASD, the recurrence risk for future sibs increased to a remarkable 30%–50%. These numbers have been verified in multiple studies [12].

As already stated, multifactorial inheritance implies both genetic and environmental factors are at work. The established heritability rates of around 0.8 or more implies that the majority of the ASD phenotype is determined by genetic factors. Still, environmental influences need to be acknowledged. The issue of environmental contributors to autism is one of the most highly debated topics in all of the body of medical literature. A discussion of this is beyond the focus of this review.

## 2. Discussion

A thorough discussion of the genetic aspects of ASDs crosses the gamut of genetic principles to be considered in human diseases and disorders. In this section we will discuss each of these parameters individually. We will also review changes in the reported data in light of changes in reported information before and after about 2008. Table 1 provides a summary of the information below.

### 2.1. Epidemiology/Population Genetics

Epidemiologic data is extremely helpful in population genetics. Knowing the specifics about occurrence, incidence, prevalence and biases of a condition is important in defining genetic factors and the magnitude of their influence on the trait. The reported overall incidence of ASDs has steadily increased over the past 3 decades. The curve has risen sharply since the year 2000. At that point, the reported incidence of ASDs was 1/150 school-aged children. In 2014 it had risen to 1/68 (a 120% increase). Boys are estimated to be affected 4–5 times as often as girls; with the reported incidence now being 1/42 in boys and 1/189 in girls. With this notable increase in reported cases, recent studies have re-evaluated the population genetics of ASDs. Some have reinforced what has already been known. Others have challenged long-standing assumptions. We will look at each of these individually.

#### 2.1.1. Recurrence Risk

Recent studies [6,13,14] have suggested that the recurrence for siblings in the situation of a single affected proband may be higher than the previously noted 3%–10%. These more recent studies reported risks of 10%–19%. While there has not been a formal weighted analysis yet of these changing numbers, these studies provide sufficient support of using an increased recurrence risk of 10%–20%. In the event of more than one affected sibling with an ASD recent studies have confirmed the estimated 30%–50% recurrence risk for subsequent sibs [14,15,16]. The newer studies have also reported relative risk ratios (RRR) for full siblings as 6.9–10.3 and significant concordance calculations with RRR estimates of 150 for monozygotic twins as compared to 8.2 for dizygotic twins [4,5].

#### 2.1.2. Heritability

Heritability is defined as the proportion of the observed phenotype that is attributable to genetic factors. Heritability estimates over the past 20 years for ASD have been in the range of 0.7 to 0.9. One recent study [4] has given a much lower estimate of 0.4–0.6. For now these data must be cautiously interpreted in light of most other reports.

#### 2.1.3. Sex Bias (Occurrence Gender)

Historically a reproducible and significant gender bias has been noted in the incidence of most neurodevelopmental disabilities. In general males have been found to have a 4–5-fold increased occurrence of these conditions [17]. This is true for ASDs. This difference is noted more so in those persons with ASD who have milder degrees of cognitive impairment. If individuals with mild cognitive impairment are considered, the gender bias is about 2 times increased in males. In individuals with more severe impairments the bias is 4–7 times increased [18]. The first assumption that would be made in this setting would be that X-linked genes might be associated with this observed increased occurrence in males. Interestingly it has been suggested that only about 10% of the reported male excess can be attributed to X-linked genes. A previously cited study [13] did not identify a gender bias. However, others have confirmed this finding [14,19]. For now it appears safe to assume that there is indeed a 4–5-fold increased incidence of ASDs in males.

#### 2.1.4. Proband Gender Effect (Recurrence)

As previously mentioned, most multi-factorial conditions show a gender bias in their occurrence. Recurrence rates can then also be shown to have a gender specific recurrence rate with the rate being higher if the affected individual is of the less frequent gender. In ASDs the recurrence rate in siblings of an affected male has been stated as 4% as compared to 7% if the affected individual is a female [3]. In more recent studies, this “proband gender effect” on recurrence in siblings has been questioned. Three studies [5,14,19] found no proband gender differences. Two other recent studies [15,20] did observe this phenomenon. From a practical standpoint, if the proband gender effect is indeed real, the small difference that would be present would not be sufficiently large enough to be predicted to be of sufficient magnitude as to affect familial decision making. Thus, while this is an interesting question and worthy of additional investigations, clinical counseling could probably be best given as 10%–20% recurrence risk for either gender.

#### 2.1.5. Reproductive Stoppage and Birth Order

Earlier observations have noted that despite a per pregnancy prediction of a 3%–10% recurrence rate of ASDs, the actual number of observed recurrences was less (2%–3%). This raised the question of reproductive “stoppage” (aka curtailment of reproduction). That is the presumption that once a couple has had a child with an ASD, they are less likely to have other children—regardless. These correlated observations suggested that stoppage might be at work, but without investigative support. Three recent reports [16,21,22] have all objectively confirmed the suspicion of stoppage in families with children with an ASD. Finally, some studies have investigated the association with birth order and ASD. Birth order is an interesting parameter to consider. In actuality birth order effects are predicted to be more of an environmental influence. Two studies have noted no birth order effect in one report [14] *versus* a decrease in ASDs in later siblings. Reported birth order effects, if real, are likely to be related in some degree to the same factors in reproductive curtailment (psycho-social environmental factors).

#### 2.1.6. Parental Age Effects

An association with an advanced paternal age (which is felt to be associated with an increased occurrence of *de novo* mutations) has previously been reported in ASDs [23]. One recent report [14] actually reported the opposite—an association with younger fathers.

**Table 1:** Summarizes the current parameters of the population aspects of ASDs.

Parameter	Value	Comments
Recurrence risk	10%–20%	Value increased based on newer studies
Relative recurrence ratio		
Monozygotic twins	150	
Dizygotic twins	8	
Full siblings	7–10	
Heritability	0.7–0.9	One recent study estimate of 0.5
Occurrence gender	4–5× higher in males	Few studies have not seen this
Proband gender effect	2× increase if female	Recent studies differ on this effect
Paternal age	Increased	One recent study saw a higher occurrence in younger fathers
Reproductive curtailment (stoppage)	Appears to be real phenomenon	
Birth order	Decreased in later sibs	To be confirmed

### 2.2. Genetic Loci and ASDs

Given the information in the preceding section, a genetic basis for ASDs is unquestionable. As such, the next question is “Can these genetic factors be identified?” In this context a couple of terms should be defined. “*Etiology*” is a specific diagnosis that can be translated into useful clinical information for the family, including providing information about prognosis, recurrence risks, and preferred modes of available therapy [24]. This definition was further refined to include the stipulation that there is “sufficient literature evidence to make a causal relationship of the disorder … and if it meets the Schaefer-Bodensteiner definition” [25]. “*Diagnostic yield*” is the proportion of cases in which the etiology can be determined after a complete evaluation. Essentially it is the batting average in finding the etiology of a given condition.

Genetic testing technologies have dramatically improved over the past 3 decades. Each significant advancement in genetic testing technology has produced an increased understanding of the genetic factors that cause ASDs. A rise in the diagnostic yield in defining an etiology for ASD parallels the introduction of these new technologies. A discussion of the causes of ASDs can be framed in the context of the ontogeny of the development of each new modality.

#### 2.2.1. Linkage

Linkage technology was developed and refined, with a further expansion into genome wide association studies. Multiple studies using this technology were reported in cohorts of patients with ASD. A summary of these results is that linkage was identified with most autosomes. A careful review of the cumulative results identified consistent reports of linkage to chromosomal regions 2q, 7q22-31 (with a parent of origin effect), 13q, 15q11-13, 16p and 17q11 (a male specific locus). Better *lod* scores were obtained if a broader phenotypic definition was used. Repeat studies with larger sample sizes did not change these results/conclusions.

Since ASDs shows a clear male predominance, a logical assumption might be that X-linked genes could be playing a major role. However, linkage studies specifically targeting the X chromosome did not identify X-linked genes as accounting for a large portion of the male predominance. Only four minor linkages to the X chromosome were noted. Stated another way, a much smaller proportion of the male preponderance is explained by X-linked genes than initially suspected [26]. This raises an interesting corollary question. If X-linked genes do not explain the male excess in cases, what does? Other possible explanations that have been proposed include (1) Mosaicism/tissue specific expression of X-linked genes; (2) Dysregulation of methylation of brain expressed genes on the X chromosome and (3) Hormonally mediated changes (*i.e.*, differential expression on the “male brain”).

#### 2.2.2. Cytogenetics

Microscopically detected chromosome abnormalities have consistently been reported in association with ASD. Refinements of chromosome analysis (prometaphase and later interphase studies) enhance detection rates. Estimated positive findings in ASDs have a reported range of 3%–12% with the best overall estimates being 3%–5% [27,28,29]. The most commonly seen abnormalities reported are deletions or duplications of the proximal 15q region. Other commonly reported aneuploidies include deletions of 2q37, 7q, 18q, and Xp and duplication of 22q13. An association with whole chromosome aneuploidies such as 47 XXY and 45X/46XY has also been seen.

#### 2.2.3. Fluorescent-*in Situ*-Hybridization (FISH)

Fluorescent-*in situ*-hybridization (FISH) technology became generally available for clinical use in the 1990s. With the introduction of FISH studies, clinical geneticists had another tool beyond just karyotype analysis for identifying mutations in patients. When applied to patients with ASDs the most common single loci FISH findings were found in association with the chromosomal regions 2q, 15q, 17p, 22q11, and 22q13.

#### 2.2.4. Chromosomal Microarray (CMA)

Around the year 2000, molecular cytogenetic techniques were introduced to clinical medicine. Chromosomal microarray (CMA) quickly became a powerful tool for identifying copy number variants (CNVs) in the human genome. When microarray studies are performed in patients with neurodevelopmental disabilities, a significant number of patients will have either small deletions or duplications identified. Currently microarray studies are still one of the highest yield diagnostic tests in these patients. In the process of the study of CNVs in ASDs, a handful of “ASD syndromes” has been identified. These syndromes are recognizable phenotypes that are associated with specific CNVs. These conditions have notable clinical features besides having ASD. Table 2 list a few of the most well-known ASD syndromes associated with small chromosomal deletions or duplications.

**Table 2:** “Autism Syndromes” Identified by Chromosomal Microarray.

Copy Number Variant	Incidence in Cohorts with ASDs	Eponym	Other Key Features (besides ASD)
1q21.1 del	1%	None	Congenital heart disease (30%)
2q22.3 del dup	<1%	Mowat-Wilson	Hirschprung disease, epilepsy, facial dysmorphisms
16p11.2 del/dup	1%	None	
17p11.2 dup	<1%	Potocki-Lupski	Hypotonia, slow growth, epilepsy
22q11.2 del	<1%	DiGeorge/Shprintzen	Multiple congenital anomalies
22q13.3 del	1%	Phelan-McDermid	Hypotonia, accelerated growth

Over the past 15 years, many studies have been published that have reported on the incidence of CNVs identified by microarrays in patients with ASDs. Cumulatively over 100 different clinically significant changes have been reported in patients with ASDs. Estimates of the prevalence of pathogenic CNVs in ASD range from 8% to 21%. Overall it has been concluded that CMA will identify a known pathogenic CNV in about 10% of all cases of ASD [12]. Prior to the introduction of microarray technology, the overall diagnostic yield for a complete evaluation of a person with ASD was accepted as being around 6%–12%. Thus the introduction of this one test modality greatly enhances the overall diagnostic yield. As will be discussed below, this high yield warrants making CMA a “first-tier” test in the etiologic evaluation of persons with ASD.

#### 2.2.5. Key ASD Loci

One of the fascinating observations that can be made from the data presented in the above section is the consistency of the findings across studies regardless of the testing modality utilized. A review of this information readily identifies several “ASD hot spots”. Specifically, certain loci jump out as consistently being identified by linkage, chromosomal studies, FISH and microarray studies. This aggregate information serves as a strong indicator for investigators that key genes that paly a strong role in the occurrence of ASD lie in these regions. Some of the strongest evidence points to strong genetic factors in the pathogenesis of ASD lie in chromosomal regions 2q, 7q, 15q11-13, 16p, 17q, 22q, Xp, and Xq. A more detailed listing of these areas of interest can be found in the ACMG practice guidelines reported in 2013 [12].

### 2.3. Clinical Genetic Evaluation of ASD

Not surprisingly, the number of requests for consultations by clinical geneticists with the referral indication of ASD has tremendously increased over the past 15 years. Of course this is in part due to the greatly increased reported incidence. In addition, there is an increased awareness among both the lay and medical communities of the recent rapid advances in genetics and genomics. As such, a large number of children with the diagnosis of ASD are being seen for an etiologic evaluation. However, many question the rationale for such evaluations. The question raised is simply “You can’t cure the condition, so what good is it in figuring out what caused it?” This is an age old question that geneticists have answered almost since the inception of the discipline. It is critically important to note that an etiologic answer can be helpful to patients and their families in many different ways [30]. Examples of these would include:

(1) Genetic counseling—including providing recurrence risk information
(2) Counseling regarding the natural history of the condition
(3) Anticipation of a later associated co-morbid condition
(4) Prevention of secondary disorders
(5) Availability of prenatal diagnosis
(6) Access to public support systems
(7) Access to syndrome-specific support groups
(8) The reassurance of knowing “Why” in reliving the stress of the unknown
(9) The possibility of a specific treatment strategy—should one be available or developed in the future

#### 2.3.1. Role of Dysmorphology/Clinical Genetics

When a patient is referred for a clinical genetic evaluation for ASD, many families want to know “Why” (discussed above) and “What will happen?” as clinical genetics is not a discipline familiar to many people [31]. A major skill set of a trained clinical geneticist is that of a “dysmorphology assessment”. The geneticist has the ability to identify differences in morphology of patients and often to assemble the list of noted findings into a recognizable pattern [32]. The identification of such patterns can then aid in directing the rest of the diagnostic workup.

When a cohort of persons with ASD is evaluated, two sub-groups are easily identified [33]. An estimated 10%–20% of persons with ASD fit into the category of “complex” or syndromic’ ASD. These are patients who, besides having ASD, are noted to have recognizable abnormalities of morphogenesis manifest as dysmorphic features, microcephaly, or cerebral dysgenesis [34]. As a whole, this group has a more guarded outcome with a higher degree of cognitive impairment and epilepsy and a greater occurrence of co-morbid medical problems. The second group (those with just ASD) has been referred to by several terms including “simple”, “essential”, “idiopathic” or “non-syndromic”. As a whole this group has a higher heritability, more affected family members, and a higher male: female ratio than the complex group [33].

The initial part of the etiologic evaluation of ASD then is to determine whether or not the patient fits into the syndromic or non-syndromic category. If it is the former the next step is to see if a specific syndromic etiology can be identified. Sometimes this can be accomplished simply as part of the initial history and physical examination. Table 3 lists some of the most important syndromes associated with ASD that should be considered. A related question in this regards is when to do an etiologic evaluation for ASD in a patient who already has a known syndromic diagnosis. For some syndromes the known association with ASD is so strong that one can safely assume that the condition is indeed the cause of the ASD. In these cases no further evaluation is indicated. For other dysmorphic syndromes the reported association is not as convincing and a full evaluation should be considered. For a listing of these two groups the reader is referred to a recent review [12].

**Table 3:** Important Dysmorphic Syndromes to Consider in the Etiologic Evaluation of ASD.

22q11.2 Deletions (Including DiGeorge and Shprintzen Syndromes)
CHARGE syndrome
Fragile X syndrome
Opitz FG syndrome
Prader Willi/Angelman syndrome
PTEN associated disorders
Rett syndrome
Smith-Magenis syndrome
Sotos syndrome

**Figure 1:**
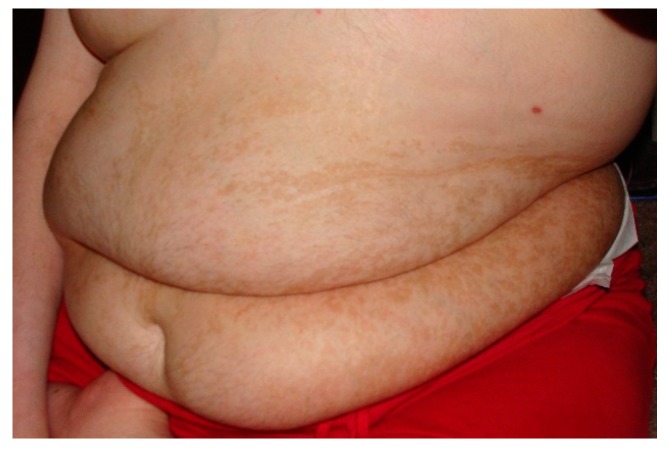
Pigmentary Changes in a Patient with Somatic Mosaicism. Note the linear pattern of the pigmentary changes.

In the discipline of dysmorphology, there is a well-known relationship between certain types of pigmentary abnormalities and neurodevelopmental disorders [35]. This association is felt to be due to the embryonic neuro-ectodermal cells that both populate the glial elements of the brain and give rise to the melanocytes in the skin. As such part of the dysmorphologic evaluation includes a careful cutaneous examination aided by the use of a Woods lamp to identify even subtle changes in skin pigmentation. In some cases abnormal pigmentation in association with ASD is indicative of a neuro-cutaneous syndrome. The strongest link here is tuberous sclerosis as a clearly identified cause of ASD [36]. Similar to the assumptions with certain dysmorphic syndromes, the co-existent diagnosis of ASD in a person with tuberous sclerosis does not warrant further investigation. Other patients may have pigmentary abnormalities noted in non-random patterns suggestive of somatic mosaicism [37]. In patients with ASD with these types of skin changes, a skin biopsy for cultured fibroblasts may be needed to identify the etiology (Figure 1).

Another important concept to consider is that of “expanded phenotypes”. An expanded phenotype refers to the full range of phenotypes seen with mutations (variants) at a specific locus (gene). What is typically seen in the progress of clinical genetics is that mutations in a gene are initially reported in association with a known clinical disorder. Soon after this relationship is defined, the same or similar mutations at the same locus are identified with different phenotypes. Over time a broad range of phenotypes associated with changes in the same gene is defined. For example, mutations in the gene MeCP2 were initially described as the cause of Rett syndrome. As the range of the expanded phenotype was defined, mutations in MeCP2 have also been seen in patients with non-syndromic cognitive impairment and cerebral palsy [38]. Salient to these discussions, 4% of females with idiopathic ASD will have pathogenic mutations in MeCP2 [12]. This highlights a couple of very important concepts. First, it must be recognized that expanded phenotypes exist and need to be appreciated in a diagnostic workup. It is not readily intuitive that MeCP2 testing should be part of an ASD evaluation. The second point is critically important: genotype does not change phenotype. If the patient with ASD is found to have a MeCP2 mutation, the diagnosis does not change to Rett syndrome, simply the cause of the ASD has been identified.

As noted in an earlier section ASD exhibits a familial pattern of multifactorial inheritance. Thus multiple members of the same kindred may be found to have ASD. Even within families, a large degree of variable expression is typically seen. Family members with ASD may differ in their degree of social and cognitive impairment. They may also differ in co-morbid medical conditions. Along the lines of the discussions of expanded phenotypes the same causative mutation may produce different phenotypes. Thus as one investigates the kindred of a person with ASD, other neurodevelopmental and neurobehavioral phenotypes need to be considered. It is not uncommon for a family with a person with ASD to also have persons affected with other conditions. The incidence of other conditions such as depression, schizophrenia, anxiety and even Attention Deficit Hyperactivity Disorder (ADHD) are higher in relatives of persons with ASD [39]. Recent discoveries of other etiologies of ASD have identified some of these links. For example, a copy number variant—deletion 17q12—has been reported with both ASD and schizophrenia in the same family [40].

An interesting question has been raised in regard to the craniofacial appearance of patients with non-syndromic ASD. Specifically, is there an “ASD face”? In a non-published series of studies, myself and one other clinical geneticist reviewed the photographs of 33 children with ASD [41]. We then cataloged any dysmorphic features noted. These findings were tabulated for recurrent features. Those features are listed in Table 4. These photographs plus another 31 photographs of age and gender matched normal children without ASD were presented to three other clinical geneticists in a blinded fashion. They were asked to identify any of the features in the established list in the 64 photographs. Five of the nine features (shortened columella, thick scalp hair, lateral extension of the eyebrows, thickened ala nasae, and prominent nasal root) occurred more often (*p* value range 0.006–0.02) in the group of children with ASDs. While this is a very interesting observation, it is not clear what, if any, clinical significance it might have.

**Table 4:** Common Facial Features Noted in Non-syndromic ASD [41].

**Thick scalp hair**
**Lateral extension of the eyebrows**
**Sloped forehead with prominent brow**
Infraorbital hypoplasia
Prominent pre-maxilla
**Short columella**
**Prominent (broad) nasal root**
**Thickened ala nasae**
Prominent philtral ridges
Small ears

Bolded features were noted to occur statistically more often in children with ASD in a blinded study.

#### 2.3.2. Guidelines for the Clinical Genetic Evaluation of ASD

With ever changing advancements in genetic testing technology comes the question of which test to use when. To answer this question the American College of Medical Genetics’ Professional Practice and Guidelines Committee developed a framework to aid the geneticists in these evaluations. The initial guidelines were published in 2008 [42] and subsequently revised [12] to reflect further changes in testing technologies and emerging findings in the literature. This report proposes a “tiered” approach to the diagnostic evaluation. The earlier tiers use testing modalities that have a higher diagnostic yield and are less invasive/easier to accomplish than others. In brief, the most recent report notes an expected diagnostic yield of 30%–40%. That is to say that a specific mono-factorial etiology can be identified in patients with ASD in 30%–40% of the cases. For details of these evaluations the reader is referred to the reference provided [12]. While these numbers may seem low to many, they represent a significant increase from the 6%–12% yield reported prior to the year 2000. As such it is recommended that every person with the diagnosis of ASD be offered a clinical genetic consultation. That is not to say that every person with ASD will receive such a work up. Many details factor into the ultimate decision of whether to proceed with testing or not. It is crucial that these families receive detailed pre-evaluation informed consent. They need to understand what the tiered process entails, what the projected costs may be (especially in light of 3rd party payer policies), any potential risks of the testing and, of course, the diagnostic yield. Once the family understands what is involved that can decide whether such an evaluation is appropriate for them.

At the time of this writing, the 2013 guidelines are only a couple of years old. However, the ever advancing science of genetic testing is soon going to necessitate another revision of these recommendations. The advent of relatively inexpensive and rapid genomic testing methods has already been applied to cohorts of patients with ASD [43,44,45,46]. Currently a handful of publications have found that whole exome sequencing (WES) and whole genome sequencing (WGS) are expected to significantly increase the diagnostic yield when applied to patients with ASD. A summary of se current reports suggests that WES/WGS may identify an additional 10%–15% of causes of ASD. It is important to note that current sequencing techniques are not very good at detecting the larger copy number variants. Thus these techniques do not obviate the use of chromosomal microarray (CMA). Microarray testing itself is a high yield test identifying an etiology of ASD in about 10% of cases. Together WES and CMA alone may identify the cause of ASD in 20% of cases [45]. Finally, it is also important to note that the diagnostic yields are different for the 2 ASD sub-groups. Not surprisingly, the yield is higher (about double) in the complex/syndromic group.

#### 2.3.3. What about Unknowns?

The recent advances in genetics and genomics have been extremely exciting and gratifying for clinical geneticists. Cases that have defied answers for literally decades are now being understood. As noted above, the increase in diagnostic yield for ASD from around 10% to almost 40% is indeed exciting. However, that still means that 60% of cases will have no answer despite a thorough evaluation. So the questions remain; “What about the unknown cases?” “What could possibly be the cause of the other cases?” We have already alluded to the fact that the application of WES/WGS testing to the diagnostic algorithm will undoubtedly increase the diagnostic yield. It is a little too early yet to definitively say by how much. However, consideration of the several early reports suggests that the application of these modalities in testing may increase the yield to 50%, maybe 60%. At that point it appears that the straight-forward, mono-factorial etiologies will mostly have been identified. The remainder of cases is likely due to more complicated mechanisms. Many such mechanisms have been suggested. Some of these would include:

(1) Mutations in non-coding DNA
(2) Epigenetic disorders including those fitting a MEGDI model—mixed genetics/epigenetics
(3) Multiple contributing loci
(4) Complex gene x gene interactions
(5) Snippets

It is highly likely that further insights into the etiology of ASD will come from investigations into these (and other) mechanisms.

### 2.4. Metabolic and Mitochondrial Disorders

Among the many genetic causes of ASD, metabolic and mitochondrial disorders deserve a separate discussion. These conditions have been the subject of vigorous debate among genetic and metabolic specialists over the past many years. The importance in sorting out the answers in these conditions lies in the commonly stated point that these conditions are “low yield, but high impact” [12]. Specifically, these conditions have the greatest potential for treatment among the myriad of causes of ASD. They may also present special situations of genetic susceptibility to environmental factors as well as project for a more progressive/deteriorative course.

Many metabolic (*i.e.*, inborn errors of metabolism) and mitochondrial conditions have been reported in association with ASD [47,48,49]. While some have been reported with the non-syndromic phenotype, many exhibit a variety of other features that stand as clues to the possible cause. Signs and symptoms that suggest such a possible etiology include poor linear growth, laboratory changes (anemia, acid-base abnormalities, lactic acidosis), cyclical vomiting, rashes, tone abnormalities, gastrointestinal problems, refractory epilepsy, changes in sensorium, neuro-regression outside of the typical speech loss of 18–24 months and microcephaly. An especially important situation to consider is developmental regression associated with fever or illness. This type of regression especially with recurrent episodes and multiple organ dysfunctions are clues to a possible mitochondrial disorder. These patients have been noted to have loss of speech after a febrile illness or immunizations with a subsequent encephalopathy. (It is particularly important to note that despite this association, immunizations do not cause ASD. There simply are no reputable studies which show any such plausible link.) In the evaluation of the etiology of a person with ASD, the presence of any of these clues should prompt an extended evaluation to include metabolic and mitochondrial causes.

## 3. Conclusions

A large body of solid and consistent studies identifies a clear genetic etiology of ASD. Like most complex phenotypes, ASD has a “multi-factorial etiology”. This means that both genetic and environmental factors contribute to the overall phenotype. Heritability estimates suggest that genetic factors by far play the largest role. Clinical genetic evaluations currently can identify the cause of ASD in 30%–40% of cases. Early evidence suggests that the addition of genomic techniques to the standardly recommended testing protocol is likely to increase this yield to over 50%. Such evaluations should be offered to families after clear and careful informed consent. Knowledge of the cause of ASD can be very empowering for families. Simply knowing “Why” can be very reassuring and directive for many families. In addition, this provides genetic information for familial risks, prognosis, and awareness of possible associated conditions.

Most importantly, the identification of a genetic etiology of ASD is the first step in establishing “personalized medicine” for these patients. What is currently quite clear is that ASD is not a single condition. It is a collective of conditions with a common, strictly defined neuro-behavioral phenotype. In reality it is “the ASDs” with literally hundreds of genetic causes identified. The delineation of specific subtypes (endo-phenotypes) of ASD will facilitate the investigation of differential responses to therapies. This coupled with a growing understanding of ASD as a disorder of the synapses should allow for the development of targeted therapies based upon a specific genetic diagnosis. While it is impossible to dogmatically state when this might happen, it is anticipated that this type of focused intervention is just on the horizon.
