# Correction: G. Bradley Schaefer. Clinical Genetic Aspects of ASD Spectrum Disorders. *Int. J. Mol. Sci.* 2016, *17*, 180

**DOI:** 10.3390/ijms17091572

**Published:** 2016-09-19

**Authors:** G. Bradley Schaefer

**Affiliations:** University of Arkansas for Medical Sciences, Arkansas Children’s Hospital, 1 Children’s Way, Slot 512-22, Little Rock, AR 72202, USA; schaefergb@uams.edu; Tel.: +1-501-364-2971; Fax: +1-501-364-1564

The author wishes to make a change to the published paper [1]. The title should read: “Clinical Genetic Aspects of Autism Spectrum Disorders”. The author apologizes for any inconvenience the change may cause.

The change does not affect the scientific results. The manuscript will be updated and the original will remain online on the article webpage.
